# Spectrum of spontaneous photon emission as a promising biophysical indicator for breast cancer research

**DOI:** 10.1038/s41598-017-13516-8

**Published:** 2017-10-12

**Authors:** Xiaolei Zhao, Meina Yang, Yong Wang, Jingxiang Pang, Eduard Van Wijk, Yanli Liu, Hua Fan, Liewei Zhang, Jinxiang Han

**Affiliations:** 1 Department of Biochemistry and Molecular Biology, Shandong University, Jinan, 250012 China; 2 Shandong Medicinal Biotechnology Center, Shandong Academy of Medical Sciences, Jinan, 250062 China; 3 Department of Neurosurgery, Shandong Cancer Hospital, Jinan, 250117 China; 4 Sino-Dutch Centre for Preventive and Personalized Medicine/Centre for Photonics of Living Systems, Leiden University, Leiden, Netherlands; 5 Meluna Research, Geldermalsen, Netherlands; 6 Department of Basic Medicine, Shandong University of Traditional Chinese Medicine, Jinan, 250355 China; 7 Shandong University of Traditional Chinese Medicine, Jinan, 250355 China

## Abstract

In this study, we investigated the spectral characteristics of Spontaneous Photon Emission (SPE) from the body surface of a human breast cancer-bearing nude mice model during the overall growth process of breast cancers. By comparing and analyzing the data, we found that there was a striking difference between tumor mice and healthy controls in the spectral distribution of SPE from the body surface of lesion site, even when the morphological changes at the lesion site were not obvious. The spectral distribution of SPE from the healthy site of the tumor mice also differed from that of the healthy controls as the breast cancer developed to a certain stage. In addition, the difference in spectrum was related with different growth states of tumors. Interestingly, there was a positive correlation between the spectral ratio (610–630/395–455 nm) and the logarithm of the tumor volume for both the lesion site (R^2^ = 0.947; *p* < 0.001) and the normal site (R^2^ = 0.892; *p* < 0.001) of the tumor mice. The results suggested that the spectrum of SPE was sensitive to changes in the tumor status.

## Introduction

Breast cancer is the most frequently diagnosed cancer and the leading cause of cancer death among females worldwide. It alone accounts for 25% of all cancer cases and 15% of all cancer deaths among females^1^. Despite increasing knowledge of the disease, its incidence is steadily increasing in many countries in South America, Africa and Asia. Breast cancer is one of the most treatable forms of cancer if it is detected and diagnosed in an early stage. Early diagnosis is also the best way to curtail the effects of the disease and improve survival^2^. As the most commonly used screening method, mammography can often detect breast cancer in an early stage, but it is imperfect. Not all breast cancers will be detected by a mammogram, especially when the morphological characteristics of the abnormal lesions are not obvious. Mammography occasionally results in false-positive results or in overdiagnosis and overtreatment of some breast cancers^1^. In addition, although the current imaging methods can provide high spatial resolution, there is relatively little information about the metabolism and molecular-level changes in the breast tissue^3–5^.

It is well known that the metabolism of breast cancer cells is different from that of healthy cells^6^. Researchers have shown increased interest in reactive oxygen species (ROS) such as hydroxyl radical (OH∙), superoxide anions (O_2_^•−^) and hydrogen peroxide (H_2_O_2_) which are by-products of cellular metabolism, and in their roles in the tumor microenvironment^7,8^. Under normal circumstances, the levels of ROS and the capacity for oxidative defenses are well balanced. However, many factors can disrupt this balance and generate excessive ROS to cause oxidative damages to biomolecules which can lead to cellular alterations and, ultimately, tumorigenesis and neoplastic transformation^8–11^. Therefore, biochemical changes occur before morphological changes in the lesion regions. In fact, ROS are now considered as a hallmark of cancer^8,11,12^. Many studies have reported that breast cancers show increased production of ROS and a high level of oxidative stress in breast cancer tissue or a significant increase in the levels of oxidative stress markers in the plasma from breast cancer patients^12–16^. In this sense, it is of great significance to develop a new sensitive and non-invasive approach to detect changes of metabolism *in vivo* to improve the detective rate and accuracy of preliminary screening of breast cancers, especially in its early stages.

Spontaneous ultra-weak photon emission (SPE), as an intrinsic attribute of biological systems, is a possible candidate for use in the development of an optical biopsy method to monitor the levels of ROS during metabolic processes in normal and abnormal cells, tissues and organisms^17–20^. SPE originates from the relaxation of electronically excited species (*e.g*., ^3^R = O*, ^1^O_2_ and ^1^P*) in biological systems. The electronically excited species come from the oxidation of lipids, proteins, and nucleic acids by ROS that forms in normal or abnormal oxidative metabolic processes^21–24^. Each electronically excited species can emit its energy as a photon at a specific wavelength. The biophysical changes accompanying dysplastic progression often lead to alterations in the optical characteristics of tissues. The SPE detection technology that is sensitive to these alterations can be used to monitor the physiological and pathological states of the biological systems. As a non-invasive biophysical marker for monitoring the state of the biological system, SPE has attracted considerable attention and has been used in many fields including agriculture^25^, food quality^26^ and healthcare^20,27–29^.

Preliminary studies have been performed to define the potential of SPE to serve as a sensitive biophysical marker to detect different physiological and pathological states of cancers based on their different oxidative metabolic states. For example, Bustamante *et al*. found a difference in the emission intensity between the logarithmic growth phase and the stationary phase using human malignant melanoma cells, and they attributed it to the difference in the activity of catalase in cells, which leaded to metabolic changes^30^. Takeda *et al*. reported the temporal changes in SPE intensities, along with cancer cell proliferation and the spectral distribution of SPE from the TE9 cancer cell line, and found that these changes in SPE were closely related to the ROS produced during the metabolic processes^31^. The experiment from Rác *et al*. illustrated that the human multiple myeloma cells U266 exposed to hydrogen peroxide would result in the formation of ^3^(R = O)* and ^1^O_2_ which lead to an immediate enhancement of the ultra-weak photon emission followed by a slow decay^32^. Kim *et al*. measured SPE from human cancerous lung tissue and adjacent normal lung tissue and the results suggested that SPE emission could be used to differentiate a tumor from adjacent normal tissue; they also showed a salient difference between squamous cell carcinoma and adenocarcinoma^18^. Chen *et al*. successfully used serum samples to distinguish patients with acute lymphoblastic leukemia from healthy volunteers^33^. In addition, a comparison between the intensities of SPE from tumor-bearing mice transplanted with ovarian cancer cells and control mice was performed by Kim *et al*.^34^. Therefore, it is expected that SPE could be a promising biophysical marker for monitoring the pathophysiological status of breast cancers.

In our previous study^35^, we demonstrated that SPE intensity from the body surface could significantly distinguish the breast cancer-bearing nude mice from healthy controls, no matter whether the morphological changes of the tumors were obvious. We also found that SPEs changed with the tumor size. In the present study, our research question focused on the spectral characteristics of SPE from the body surface of a human breast cancer-bearing nude mice model and healthy controls during the entire breast cancers growth process. We hope to explore the relationship between the spectral components of SPE and the tumor stages more deeply. In this way, we could provide a more precise evidence for the use of SPE as a non-invasive biophysical indicator in the breast cancer research.

## Results

### Performance of the two PMT in the detection system

**Figure 1:**
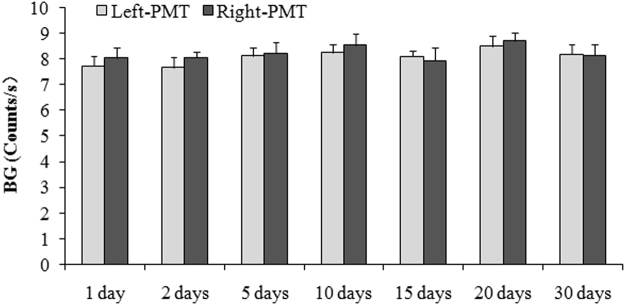
BG signals of the left and right PMT without filters at the same time (2:00 p.m.) on different days.

Fig. 1 displayed the BG signals of the left and right PMT without filters at the same time (2:00 p.m.) on different days. The figure shows that the performance of the detection system was stable, and there were no significant differences between the left- and right-PMT.

### Spectral features of SPE from the body surface of mice in each group throughout the growth process of breast cancer

**Figure 2:**
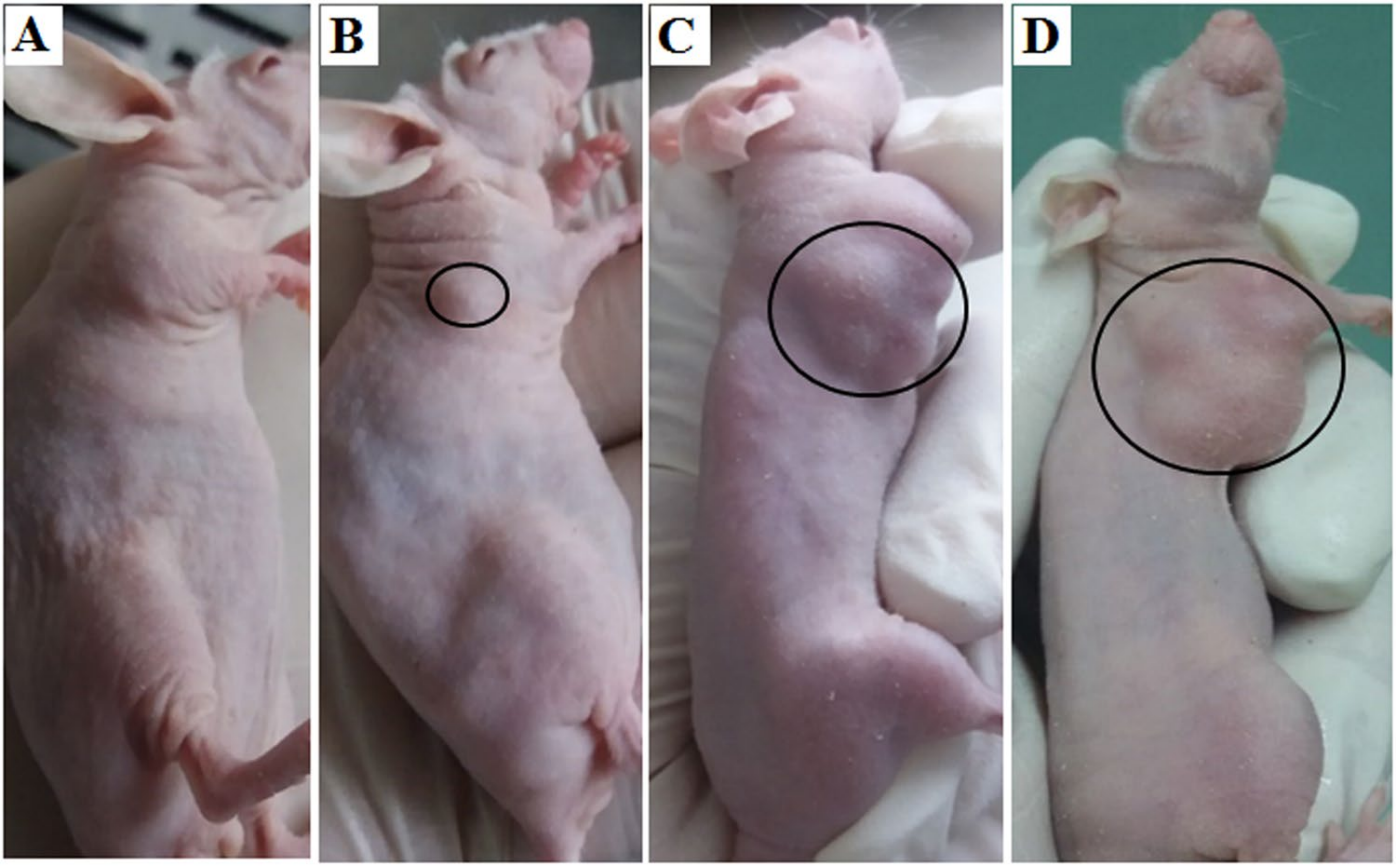
Different growth stages of a mouse whose right axillary was injected with breast cancer cells. (**A**) Incubation period of breast cancer; (**B**) Tumor diameter less than 0.5 cm; (**C**) Tumor diameter between 1 and 1.5 cm; (**D**) Tumor diameter greater than 1.5 cm.

Forty nude mice were used in this study: twenty-five in experiment group, ten in the control group and five in the normal group. From the twenty-five mice in the experiment group, twenty-one mice grew tumors (labeled as tumor mice group), but the other four mice did not (labeled as tumor-free mice group). Figure 2 shows an example of the different growth stages of a mouse whose right axillary was injected with breast cancer cells.

**Figure 3:**
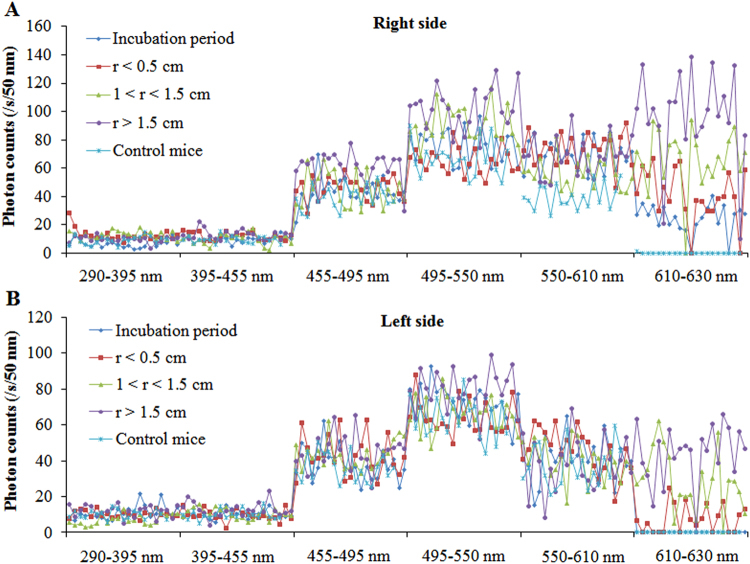
The spectral distributions of SPE from both sides of twenty-one tumor mice in different growth stages of breast cancers and nineteen healthy controls. (**A**) Spectral distributions on right sites of tumor mice in different growth stages of cancers and healthy controls. (**B**) Spectral distributions on left sites of tumor mice in different growth stages of cancers and healthy controls. r, diameter of tumor.

To analyze the spectral features of SPE from the body surface of mice in each group with respect to different growth stages of the tumor, measurements began when the breast cancer cells were in the incubation period and continued twice a week until the tumors were obvious. The detailed measurement protocol is described in the section of measurement method. The photon data (with different cut-off filters) for both sides of each mouse from each group at different growth stages of tumor (as shown in Fig. 3) were recorded, and were averaged in mean ± standard deviation (SD) corrected for their own BG signals. The subsequent calculation method was the same as that reported by Van Wijk^36^. The difference in SPE intensity between two filters with successive wavelength cut-offs was used to calculate the photon emission of a particular wavelength range. The photon emission of the particular wavelength range was mathematically corrected for the transparency of the filters and the quantum efficiency of the PMT. The detailed quantum efficiencies of corresponding particular wavelength range of the used PMT’s are described in the “Methods” section. If the difference was not significant (*p* > 0.05), the photon emission of the mouse with that wavelength range was considered to be zero. The final estimation of the spectral distribution of each side of a mouse was expressed in cps per 50 nm. Because of the similar features (*p* > 0.05) of spontaneous photon emission among tumor-free mice, normal mice, and controls which were consistent with our previous study^35^, we merged them into one group: namely, the control group. The spectral distributions of SPE from the body surface of twenty-one tumor mice and nineteen control mice throughout the growth process of the breast cancers are illustrated in Fig. 3.

**Figure 4:**
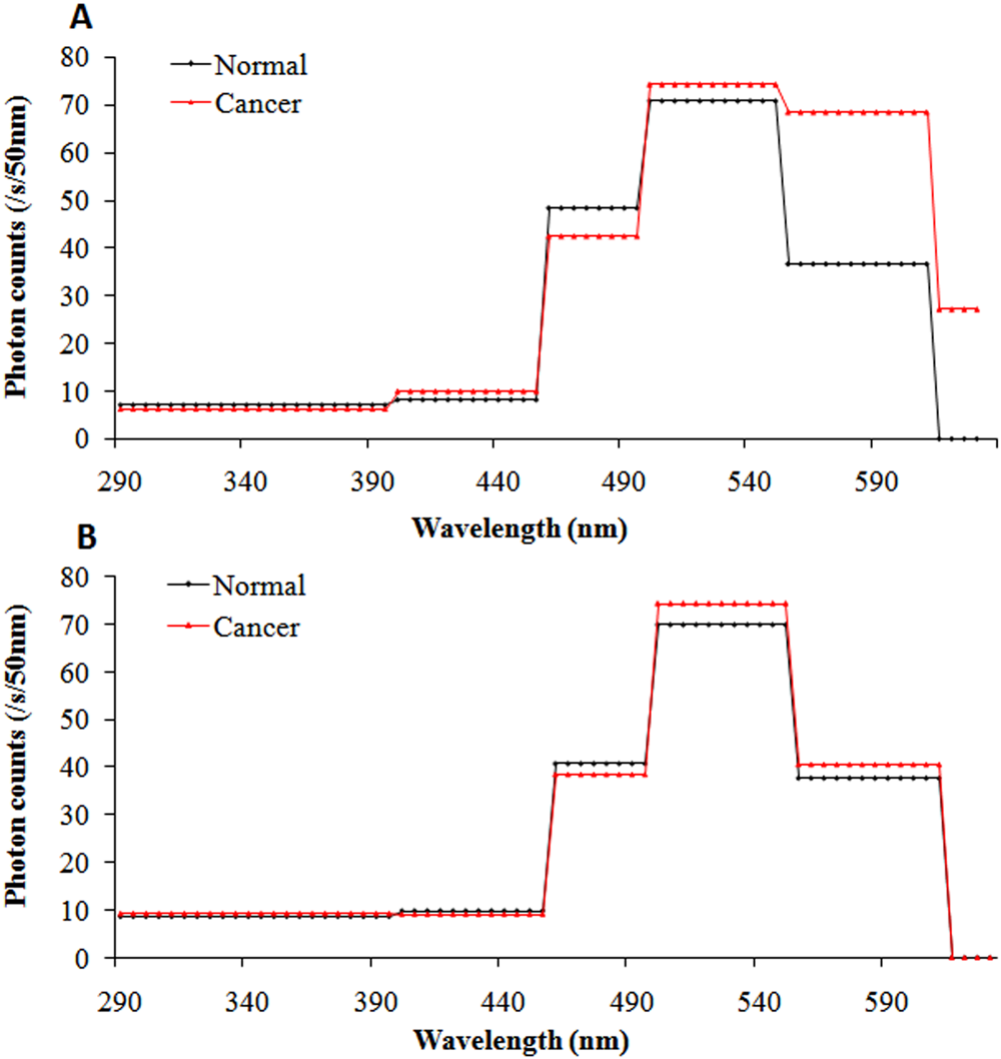
Spectral distribution of SPE from both sides of tumor mice and controls in the incubation period of breast cancers. (**A**) Spectral distribution on right lesion sites of tumor mice (red line) and same sites on controls (black line). (**B**) Spectral distribution on left sites, which corresponded to lesion sites of right sides of tumor mice (red line) and the same sites for controls (black line).

**Figure 5:**
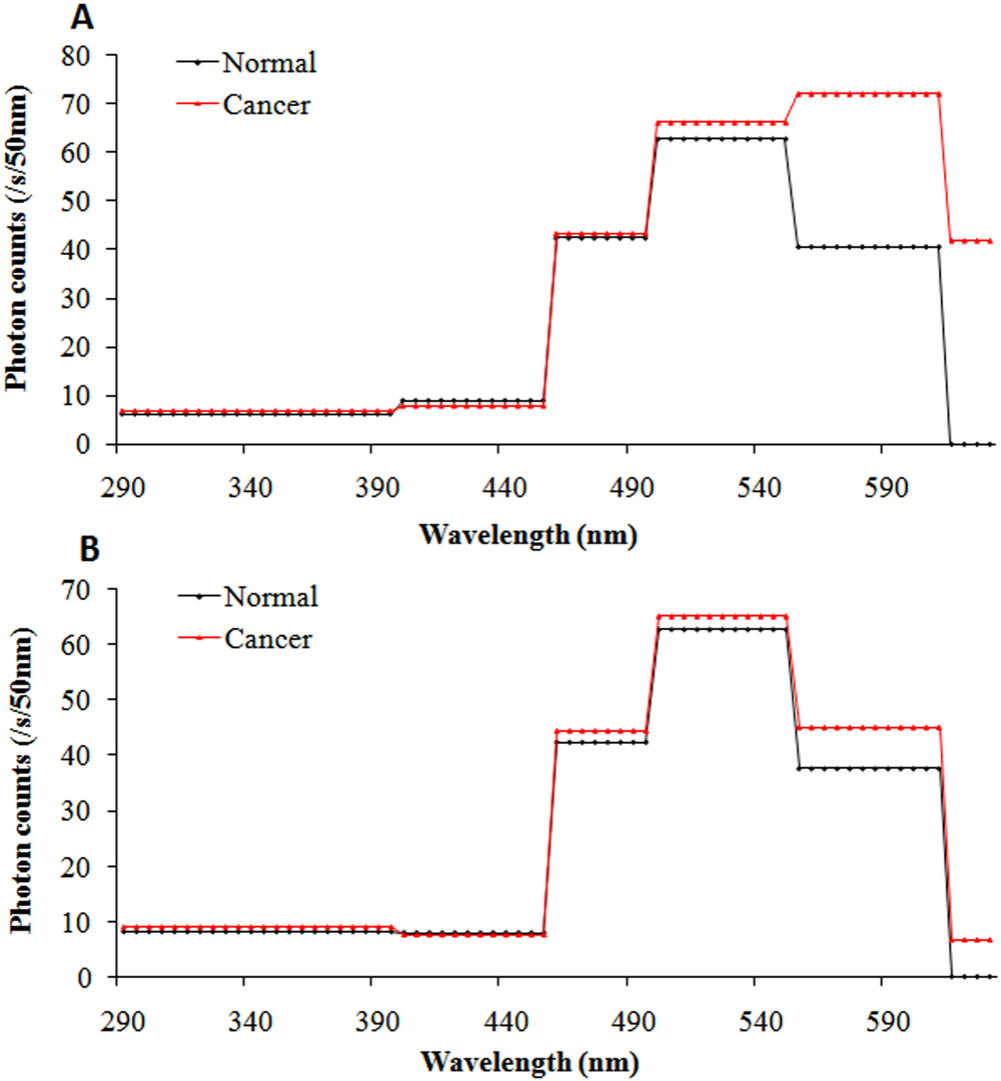
Spectral distribution of SPE from both sides of tumor mice and controls when tumor diameters were less than 0.5 cm. (**A**) Spectral distribution on right lesion sites in tumor mice (red line) and same sites in controls (black line). (**B**) Spectral distribution on left sites, which corresponded to the lesion sites of right sides in tumor mice (red line) and same sites in controls (black line).

**Figure 6:**
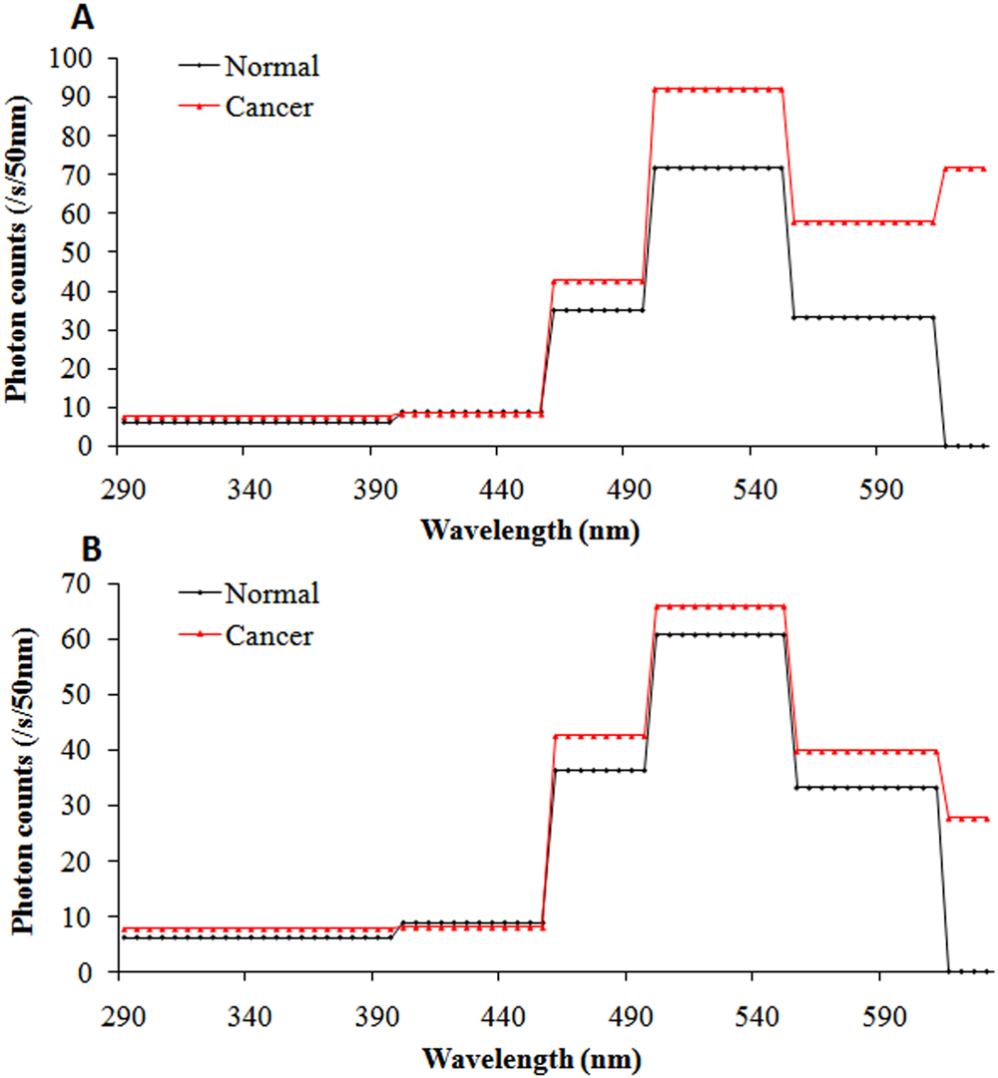
Spectral distribution of SPE from both sides of tumor mice and controls when the tumor diameters were larger than 1 cm and less than 1.5 cm. (**A**) Spectral distribution on right lesion sites in tumor mice (red line) and same sites in controls (black line). (**B**) Spectral distribution on left sites, which correspond to the lesion sites of right sides in tumor mice (red line) and same sites in controls (black line).

**Figure 7:**
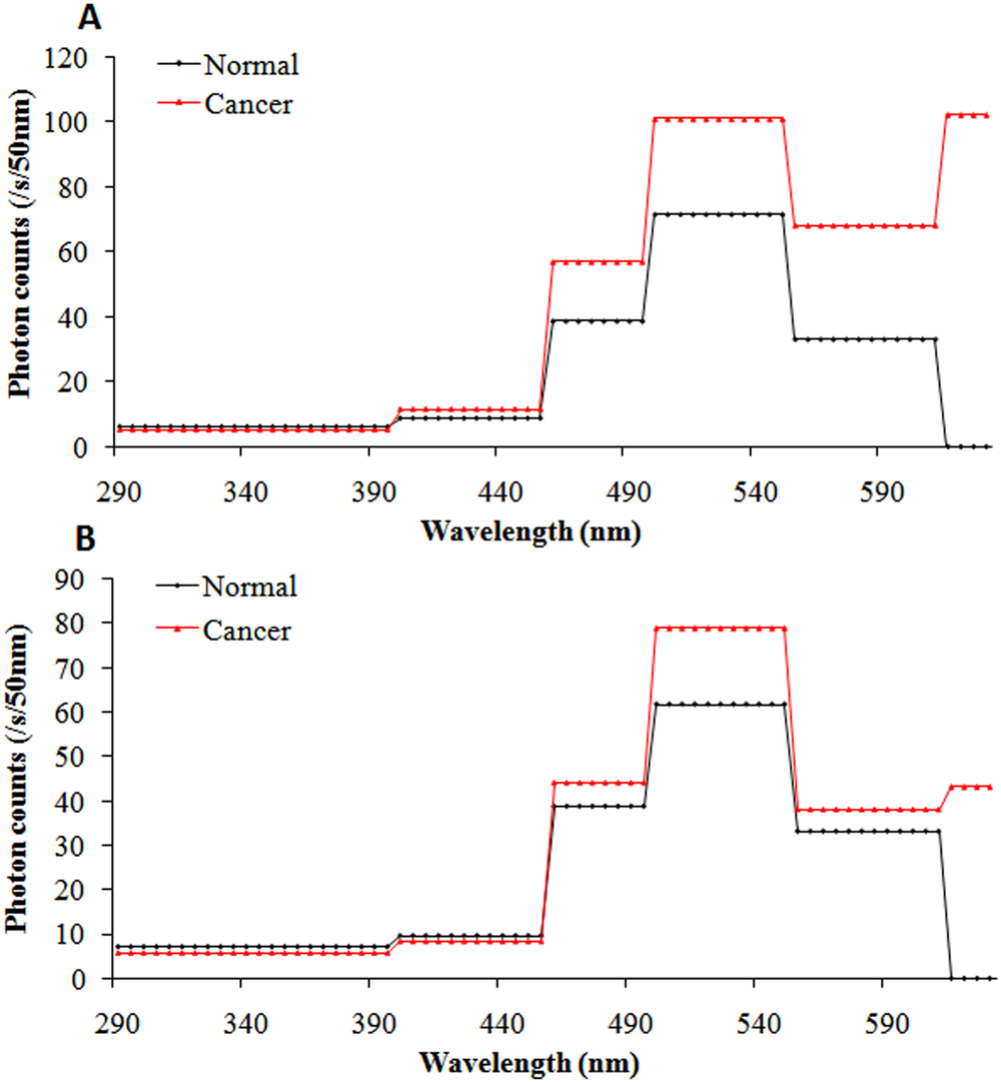
SPE spectrum from both sides of tumor mice and controls when tumor diameters were larger than 1.5 cm. (**A**) Spectral distribution on right lesion sites in tumor mice (red line) and same sites in controls (black line). (**B**) Spectral distribution on left sites, which corresponded to lesion sites of right sides in tumor mice (red line) and same sites in controls (black line).

The data in Fig. 3 illustrates that there is a high individual variance of spectral distribution between different mice. However, the trend of spectrum changes in different mice in the same groups was similar. In order to clearly exhibit the spectral characteristics of SPE during the tumor growth process, we averaged the spectral data of nude mice at the same growth stage of tumors from the same group. The results were displayed in Table 1 and Figs 4–7.

**Table 1:** The average signal intensity of SPE at different wavelength range from twenty-one tumor mice in different cancer growth stages and nineteen healthy controls. r, diameter of tumor.

Wavelength range	Incubation period	r < 0.5 cm	1 < r < 1.5 cm	r > 1.5 cm	Control mice
Right side of mice					
290–395 nm	6.84 ± 2.91	7.40 ± 4.41	7.91 ± 2.94	5.22 ± 2.27	6.33 ± 2.89
395–455 nm	9.76 ± 2.19	7.35 ± 2.67	8.70 ± 3.98	9.70 ± 3.43	8.54 ± 2.49
455–495 nm	42.07 ± 9.27	43.04 ± 8.22	42.32 ± 12.76	57.45 ± 11.27*	38.46 ± 8.36
495–550 nm	74.04 ± 11.84	64.47 ± 9.19	91.02 ± 12.54*	101.41 ± 14.81*	66.72 ± 11.97
550–610 nm	68.98 ± 11.26*	71.09 ± 11.23*	57.11 ± 11.95*	68.69 ± 13.76*	38.24 ± 8.85
610–630 nm	26.51 ± 10.33*	41.20 ± 18.41*	79.73 ± 21.11*	105.86 ± 32.66*	0.00
Left side of mice					
290–395 nm	7.10 ± 4.17	8.19 ± 1.96	7.02 ± 3.06	6.01 ± 3.24	7.06 ± 2.48
395–455 nm	9.20 ± 2.39	8.33 ± 3.17	8.45 ± 2.94	8.71 ± 4.02	9.05 ± 2.78
455–495 nm	38.06 ± 9.35	44.88 ± 11.23	43.41 ± 7.85	44.80 ± 9.84	36.01 ± 7.85
495–550 nm	72.21 ± 9.68	67.94 ± 9.54	69.85 ± 9.53	79.61 ± 11.57*	63.95 ± 10.98
550–610 nm	41.45 ± 12.10	43.09 ± 12.02	39.76 ± 10.83	37.78 ± 14.83	34.79 ± 11.51
610–630 nm	0.00	7.49 ± 7.74*	28.33 ± 16.75*	43.80 ± 16.40*	0.00

*Statistically significant compared with control mice.

As depicted in Fig. 4, the spectral distribution of SPE from the right and left sides of control mice was similar: the maximal peak was 495–550 nm, and the photon signals from both sides were no longer detectable with cut-off filters at 610 nm and higher. The spectral distribution of SPE from the left side (no tumor side) of tumor mice was consistent with that from control mice, whereas the spectral features of SPE from the right side (tumor transplantation side) of tumor mice was different. For the right side of tumor mice, the wavelength range of the maximal peak was the same as that from the controls; however, the signal at 550–610 nm was 1.9-fold higher than that of controls. In addition, the spectral distribution of the right side of tumor mice showed relatively high emission at 610–630 nm, contrary to the zero emission of the right side of controls. This illustrated that the spectrum of SPE from the tumor transplantation site of mice surface had already changed in the incubation period of breast cancer (before any morphological changes) in the tumor mice.

Figure 5 illustrates the spectrum of SPE from both sides of tumor mice and controls when the tumor diameters were less than 0.5 cm. There were no significant differences between the tumor mice and controls in the spectral feature of SPE from the left side (normal side) at this tumor stage (r < 0.5 cm), except for some photon signals at 610–630 nm in tumor mice. However, from the right side (tumor transplantation site) of the tumor mice, the maximal peak of spectral distribution appeared at 550–610 nm, which was distinctly different from both the controls and the tumor mice when the tumor was not obvious. Compared with the tumor mice during the incubation period for the breast cancers, the photon signals from the tumor mice at this tumor stage increased significantly (approximately 1.7-fold) at 610–630 nm. In other spectral components of SPE, the difference between the right sides of tumor mice and the controls was small.

Distinct from the tumor mice data mentioned above, two peaks appeared in the spectrum of SPE from the right sides of tumor mice when the tumor diameter was larger than 1 cm and less than 1.5 cm as shown in Fig. 6A: one peak appeared at 455–495 nm, which was the same as that from the right side of the controls, and another peak appeared at 610–630 nm. Notably, at 610–630 nm in the spectrum of SPE (shown in Fig. 6B), the emission levels from the left side of tumor mice at this tumor stage were 4-fold higher than the emission from the left side of tumor mice when tumor diameters were less than 0.5 cm.

In Fig. 7A, SPE from the right side of tumor mice at this tumor stage (r > 1.5 cm) showed similar spectral features with that from the same side of tumor mice when tumor diameter was between 1 cm and 1.5 cm. However, compared with the spectrum displayed in Fig. 6A, the photon intensities of SPE from the tumor mice at this stage were significantly increased at 455–495 nm and 610–630 nm. Interestingly, the distribution of SPE from the left side of tumor mice at this tumor stage, as shown in Fig. 7B, included a small peak that appeared at 610–630 nm, and the signals at 495–550 nm tend to increase compared to the spectrum in Fig. 6B.

**Figure 8:**
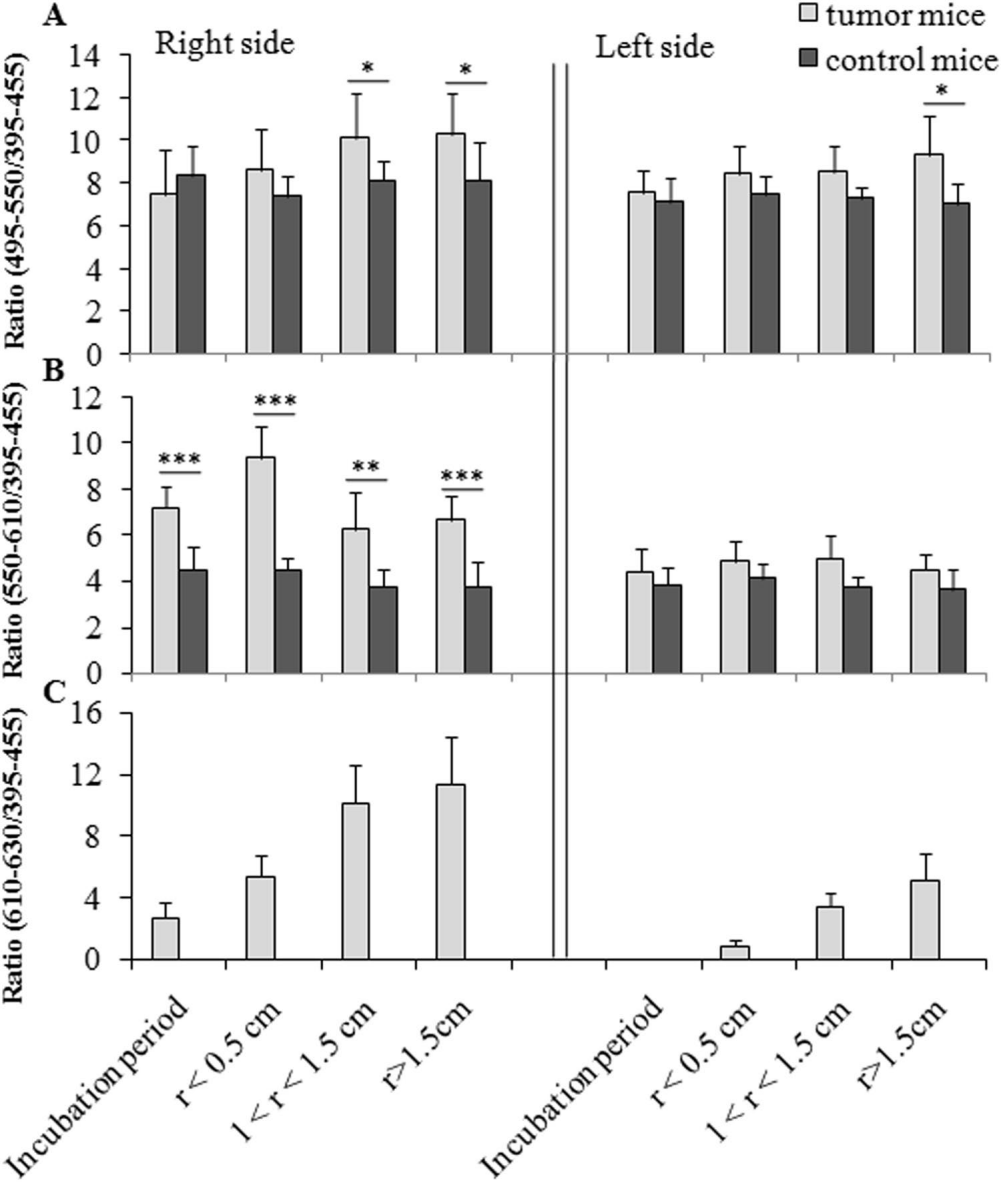
Ratio between various spectral components and component at 395–455 nm for tumor mice and controls at different growth stages of cancers. r, diameter of tumor. *p < 0.05, **p < 0.01, and ***p < 0.001.

To eliminate individual differences and obtain more accurate data for further exploration of the spectral characteristics of SPE during the tumor growth process, we calculated the ratio between various spectral components (495–550 nm, 550–610 nm, and 610–630 nm) and the component at 395–455 nm for tumor mice and controls in different cancer growth stages. The data are presented in Fig. 8.

From the results in Fig. 8, it can be concluded that the growth of cancer cells influenced the spectral distribution of SPE of the mouse body surface during the change in tumor size. The spectral components of SPE at range 550–610 nm and 610–630 nm even changed before any morphological changes at the transplantation site were visible, as shown in Fig. 8B–C. The data shown in Fig. 8A and C illustrated that, the spectral components of SPE at 495–550 nm and 610–630 nm from the healthy site (left side) of tumor mice also changed when cancers developed to a certain stage, particularly at 610–630 nm. The ratio between 610–630 nm and 395–455 nm tended to increase with increasing tumor size for both the right and left sides of the tumor mouse, as displayed in Fig. 8C, but this feature was not observed in other ratios. These differences could be used to discriminate tumor mice from healthy mice, even if tumors were not obvious and to identify the growth stages of cancers to a certain extent.

### Correlation analysis between ratio (610–630/395–455 nm) and tumor volume

**Figure 9:**
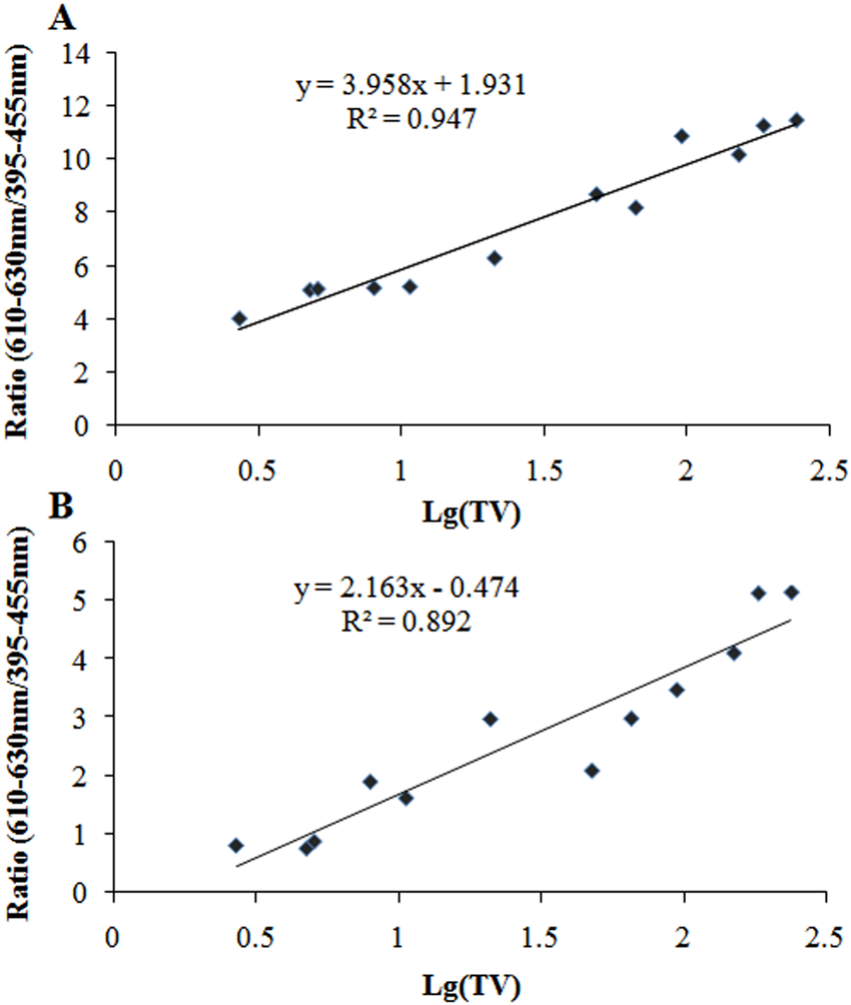
Correlation analysis between ratio of spectral components (610–630/395–455 nm) of SPE from right (**A**) and left side (**B**) of tumor mice and their tumor volume. Lg(TV), the logarithm of tumor volume; the unit is mm^3^. *P* < 0.001 for both A and B.

Following the result in Fig. 8C, we analyzed the correlation between the ratio of spectral components (610–630/395–455 nm) and tumor volume. The results are depicted in Fig. 9.

The results displayed in Fig. 9 suggested that, the spectral ratio between the wavelength ranges 610–630 nm and 395–455 nm was positively correlated with the logarithm of tumor volume for both the right side (R^2^ = 0.947; *p* < 0.001) and left side (R^2^ = 0.892; *p* < 0.001) of the tumor mice, although the correlation coefficient for the left side was relatively low.

## Discussion

It is known that metabolic processes are the fundamental biochemical reactions in biological system and result in the generation of ROS, which is known to play an important role in the formation of electronically excited species such as triplet carbonyls (^3^R = O*), excited pigments (P*) and singlet oxygen (^1^O_2_). SPE originates from the relaxation of these electronically excited species, and each electronically excited species can emit its energy as a photon at a particular wavelength. Photons from excited carbonyl groups have wavelengths in the near UVA and blue-green regions (350–550 nm), singlet (^1^P*) and triplet excited pigments (^3^P*) appear in the green-red (550–750 nm) and red-near IR (750–1000 nm) regions, and singlet oxygen is observed in the red (634 nm and 703 nm) and near IR (1270 nm) regions^23^. In animal cells, the generation of ROS is related to enzymatic reactions in mitochondria, cytoplasm and peroxisomes, and cellular respiration in mitochondria is a major source^37–39^. When metabolic processes change during dysplastic progression, the type and number of electronically excited species change accordingly, leading to alterations in the optical characteristics of cells, tissues and organisms. SPE detection technology that is sensitive to these alterations could be used to reflect the underlying metabolic processes in cells and monitor the physiological and pathological states of the biological systems from an optical perspective.

Unlike the normal cells, which primarily use oxidative phosphorylation as an energy source under aerobic conditions and glycolysis under anoxia, cancer cells use glycolysis as their major energy source regardless of whether oxygen levels are sufficient^40–43^. This difference in cellular metabolism leads to changes in several related biochemical reactions, which result in changes in ROS levels of the cancer cells. Accordingly, the SPE from cancer cells differ from those of healthy cells as we described in the introduction. However, most research has focused on tumor cells or tissues, and few studies have investigated the *in vivo* characteristics of SPE from subjects with cancer.

In our previous study, we found that SPE intensity from the body surface could significantly distinguish breast cancer-bearing nude mice from controls, regardless of whether morphological changes in tumors were obvious, and SPE changed with the tumor size^35^. In this paper, we further researched the spectral characteristics of SPE from the body surface of human breast cancer-bearing mice and healthy controls during the entire process of breast cancer growth to explore the relationship between the spectral components of SPE and the tumor stages more deeply. Our results displayed a remarkable difference in the spectral distribution of SPE between the tumor mice and controls. In the control mice, the maximum peak of the spectral distribution of SPE was 495–550 nm for both the right and left sides of the mice. In the tumor mice, the spectrum of SPE from the body surface changed with tumor stage.

For the spectrum of SPE from the tumor transplantation side of tumor mice in the incubation period of tumor, although the spectral maximum peak was still at 495–550 nm, the signal levels at 550–610 nm and 610–630 nm significantly increased (*p* < 0.001) compared to control mice. When the tumor was obvious but diameter less than 0.5 cm, the spectral maximum peak showed a substantial shift towards the red part of the spectrum from 450–550 nm to 550–610 nm, also the signal levels at 610–630 nm increased. In addition, the signal levels at 495–550 nm and 610–630 nm increased even more when the tumor size further increased, and two peaks appeared in the spectrum of SPE: one at 495–550 nm, as in the controls; and another at 610–630 nm. Some researchers reported that the skin melanin might have some effects on SPE^44,45^. Based on it, we performed a melanin analysis of skin from the nude mice using the Masson-Fontana silver staining method. We found that the skin melanin distribution in the nude mice showed negative results (see Supplementary Fig. S1). These results suggested that the skin melanin in nude mice was deficient and had no effect on SPE from the body surface of nude mice. Compared to the specific wavelength emitted by the electronically excited species, we speculated that changes in triplet excited carbonyl (^3^R = O*), singlet excited pigment (^1^P*) and singlet oxygen (^1^O_2_), which are involved in a series of complex redox reactions, maight be mainly responsible for the changes in spectral distribution of SPE from the right side of tumor mice. The increase in blood vessels at the lesion site and changes of active components in blood caused by cancers may also impact the spectral distribution of SPE, to a certain extent. In addition, with the growth of cancers, the signal intensities at 495–630 nm showed an increasing trend, especially at 610–630 nm. Although the precise mechanism by which ROS affects the spectrum of SPE is not clear, our results indicate that the spectrum of SPE is sensitive to the changes in physiological and pathological conditions of lesion site and could be used as a meaningful biophysical indicator for tumor analysis in breast cancer, even if the cancer is in its primary stage.

For SPE from the no tumor side of tumor mice compared to controls, their spectral features were similar when the tumor diameter was less than 0.5 cm. With further tumor growth, the signal intensities at 610–630 nm subsequently increased; when the tumor diameter was larger than 1.5 cm, the SPE spectrum showed a second small peak at 610–630 nm, and the signal levels significantly increased at 495–550 nm (*p* = 0.016). These results may be related to changes in type and number of electronically excited species in blood or due to the tumor metastasis. Several researchers have demonstrated that the rate of singlet oxygen and hydrogen peroxide production was measurably higher in the plasma of breast cancer patients than in controls^14,46^. Nevertheless, further studies are needed.

Another interesting finding in this study was that, the spectral ratio between the wavelength range 610–630 nm and 395–455 nm was positively correlated with the logarithm of the tumor volume for both the right side (R^2^ = 0.947; *p* < 0.001) and the left side (R^2^ = 0.892; *p* < 0.001) of the tumor mice, as showed in Fig. 9. This indicates that the spectral components of SPE from the tumor mice have a close relationship with the tumor status.

## Conclusion

We reported the spectral characteristics of SPE from the body surface of human breast cancer-bearing nude mice models throughout the breast cancer growth process by using a high-sensitivity double-PMT SPE-detection system and a series of cut-off filters. Our data showed that the spectral distribution of SPE from the lesion side of the body surface of tumor mice was significantly different from that from the same side of healthy controls, regardless of whether there were visible morphological changes at the lesion site. As breast cancer developed (1 < r < 1.5 cm in this paper), the spectral distribution of SPE from the healthy side of the tumor mice also differed from the controls. In addition, the difference in spectrum was related with different growth states of tumors. Interestingly, there was a positive correlation between the spectral ratio (610–630/395–455 nm) and the logarithm of the tumor volume for both the right and left sides of the tumor mice. Although the precise mechanism of SPE from the body surface of nude mice has not been elucidated fully, these results indicate that the spectrum of SPE from the body surface contain abundant metabolic information and is closely related to breast cancer growth. The relationship between the ROS at the lesion site or serum at different growth stages of tumors and the spectral components of SPE from the body surface should be studied next. In our opinion, these first preliminary results are interesting. However, further data acquisition is necessary to come to the conclusion that SPE spectra might be a potential biophysical indicator for breast cancers research, particularly in their early stages.

## Methods

### Experimental detection system

**Figure 10:**
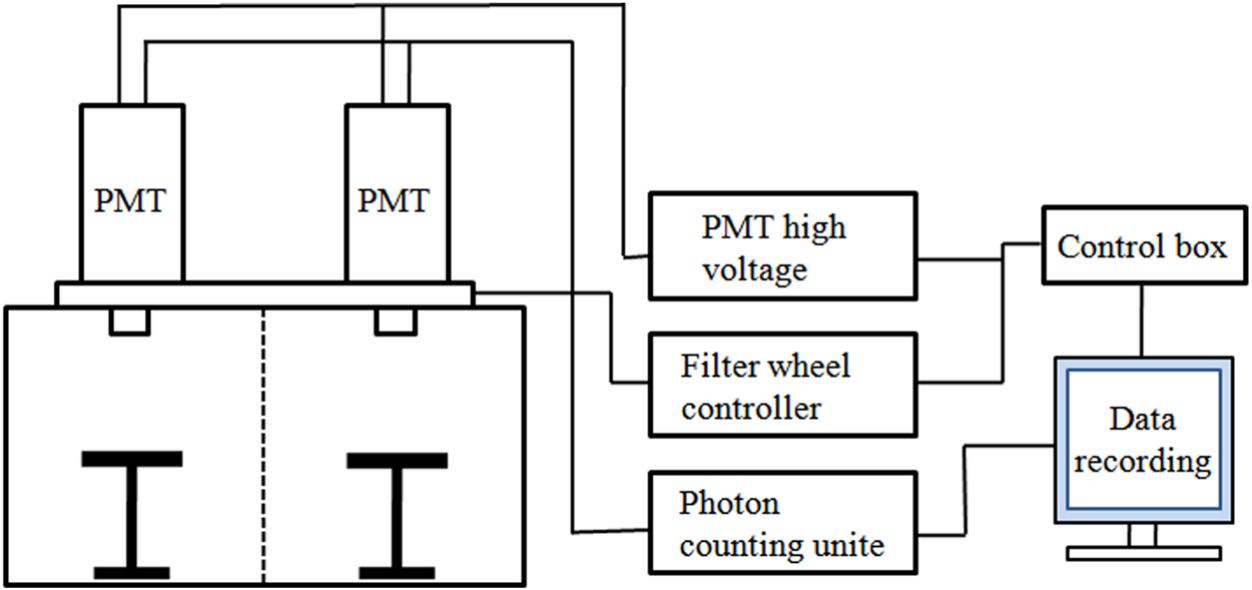
Schematic representation of the double-PMT SPE-detection system used in this study.

A schematic representation of the double-PMT SPE-detection system is illustrated in Fig. 10. The main components of the detection system include two PMT (ET Enterprises, Britain, 9235QA), a high voltage power supply (Sens Tech PM20), a filter wheel and shutter system, two photon-counting units (C9744), a dark box consisting of two chambers, a control box and a computer including measuring and photon-counting software. The system was placed in a special operation room to ensure magnetic shielding. Two PMTs sensitive in the spectral of 290–630 nm were installed on the top of the two chambers of the dark box. In front of the PMT, a filter wheel system was placed. Spectral analysis was performed using a set of filters (Schott Glaswerke AG, Main, Germany) with cut-off wavelengths at 395 nm, 455 nm, 495 nm, 550 nm and 610 nm, respectively. In addition to the above filters, one opening without filter was included in the filter wheel. The quantum efficiencies of the PMT for different spectral bands were 27% (290–395 nm), 23% (395–455 nm), 15% (455–495 nm), 6% (495–550 nm), 3% (550–610 nm) and 2% (610–630 nm).

To position the mice and ensure that the distance between the detection site and the PMT window was the same (1.0 cm) for all measurements, a sample holder with adjustable height was placed in each chamber of the dark box. The special design of two chambers and two PMT in a single dark box allowed us to measure two mice simultaneously. Temperature in the operation room was controlled at 25 ± 1 °C.

### Cell lines

The human breast cancer cell line MDA-MB-231 used in this study was purchased from the cell bank of the Shanghai Institute of Cell Biology, Chinese Academy of Sciences. The cells were cultured in DMEM supplemented with 100 U/mL penicillin, 100 µg/mL streptomycin and 10% FBS, and incubated at 37 °C in a humidified atmosphere of 5% CO_2_ in air as a monolayer culture in plastic culture plates (100 mm diameter). The cells were routinely subcultured when 80% confluence was reached.

### Subcutaneous xenograft transplantation

Forty female BALB/C nude mice (5 weeks old) were obtained from the Beijing HFK Bioscience Company and were randomized into three groups: normal group, n = 5; control group, n = 10; and experiment group, n = 25. The MDA-MB-231 cells with good growth were trypsinized and suspended in physiological saline. In the experimental group, a total of 5 × 10^6^ cells in 0.15 mL of physiological saline were injected subcutaneously into the right axillary of each nude mouse after one week of acclimation. For the control mice, their right axillary was injected with 0.15 mL of physiological saline only. The normal mice remained free from any injections. After injection, the mice were kept in a pathogen-free isolation facility under a 12-h light/dark cycle at 22–24 °C and 50% humidity with food and water available ad libitum. The animal studies were approved by the Animal Care and Ethics Committee of Department of Medicine and Life Sciences at the Shandong Academy of Medical Science and performed in accordance with the relevant guidelines and regulations.

### Measurement method

Each test was conducted between 2 p.m. and 4 p.m. to reduce the influence of diurnal rhythms^47^. To ensure the consistency of the experiment, the following protocol was observed for all measurements. **a):** Mice were weighted and anesthetized using 7% pentobarbital sodium (70 mg/kg) to keep animals in exactly the same position during measurement. After injection, the mice were held in a cage for 5 min, and then placed in a completely dark room with controlled temperature (25 ± 1 °C) and humidity (50%) 20 min before the start of the measurement to eliminate the influence of external light. **b):** The empty chamber signals (BG) without and with filters in a sequence from 395 nm to 610 nm in both PMT were measured for 3 min at intervals of 1 s before measuring the mice to ensure the detection performance of the experimental system and for follow-up data analysis. **c):** Subsequently, two pretreated mice were placed on the two sample holders in different chambers, respectively. In this way, we gained signals from two mice, synchronously. The signals from the tumor transplantation site on the right sides of the two mice and the same site on the left sides were sequentially recorded. The duration for each filter was 3 min, with an interval time of 1 s, and the recording time for each side of a mouse with a full set of filters took approximately 18 min; roughly 40 min was required for a complete measurement. The next measurement was performed immediately thereafter. **d):** Finally, the BG without and with filters was measured again. It should be emphasized that despite the small difference between the two PMTs, our experimental data showed that the difference in the signals obtained by the two PMT from the same mouse were not significant.

Measurement of tumor transplantation sites began when the breast cancer cells were in the incubation period, and measurements were performed twice per week until tumors were obvious (as shown in Fig. 2).

### Data analysis

Statistical analysis of the data was performed with SPSS 18.0 (SPSS, USA). The differences were considered significant at *p* < 0.05. Data calculations and graphing were performed using Origin 9.1. Pearson correlation analysis was used to evaluate the correlation between SPE and the tumor volume (TV), and the TV was calculated by the following formula: TV = 1/2ab^2^, where “a” represents the length of the tumor and “b” represents the width of it.

### Data Availability

The datasets generated during and/or analysed during the current study are available from the corresponding author on reasonable request.

## Electronic supplementary material

Supplementary Information
