# Supplementary material for: Spectrum of spontaneous photon emission as a promising biophysical indicator for breast cancer research
Source: Sci Rep. 2017 Oct 12;7:13083. doi: 10.1038/s41598-017-13516-8 (PMC5638945; doi:10.1038/s41598-017-13516-8)
Supplement: Supplementary file 1 — Supplementary Information [file 41598_2017_13516_MOESM1_ESM.pdf]

Spectrum of spontaneous photon emission as a promising biophysical indicator for breast cancer research

Xiaolei Zhao[1], Meina Yang[2], Yong Wang[3], Jingxiang Pang[2], Eduard Van Wijk[4,5], Yanli Liu[6], Hua Fan[2], Liewei Zhang[7], Jinxiang Han[1,2]*

[1]Department of Biochemistry and Molecular Biology, Shandong University, Jinan 250012, China. [2]Shandong Medicinal Biotechnology Center, Shandong Academy of Medical Sciences, Jinan 250062, China. [3]Department of Neurosurgery, Shandong Cancer Hospital, Jinan 250117, China. [4]Sino-Dutch Centre for Preventive and Personalized Medicine/Centre for Photonics of Living Systems, Leiden University, Leiden, Netherlands. [5]Meluna Research, Geldermalsen, Netherlands. [6]Department of Basic Medicine, Shandong University of Traditional Chinese Medicine, Jinan 250355, China. [7]Shandong University of Traditional Chinese Medicine, Jinan 250355, China. Correspondence and requests for materials should be addressed to J.X.H. (email: samsjxhyx@163.com)

Melanin analysis of skin from a nude mouse

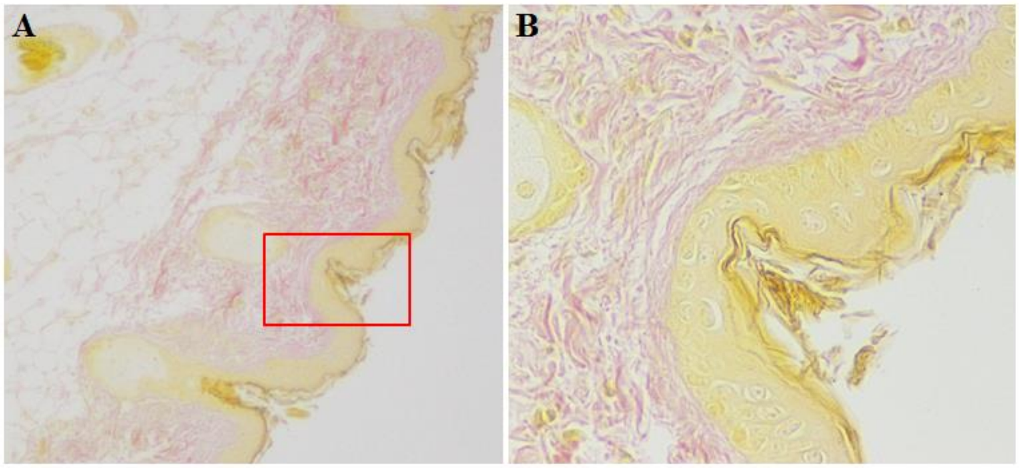

Supplementary Figure S1. The melanin distribution in the skin of a nude mouse. A, 100×. B, 400×.
